# Differentiation of two human neuroblastoma cell lines alters SV2 expression patterns

**DOI:** 10.1186/s11658-020-00243-8

**Published:** 2021-02-15

**Authors:** Emilia Lekholm, Mikaela M. Ceder, Erica C. Forsberg, Helgi B. Schiöth, Robert Fredriksson

**Affiliations:** 1 Functional Pharmacology, Department of Neuroscience, Uppsala University, Uppsala, Sweden; 2 Molecular Neuropharmacology, Department of Pharmaceutical Biosciences, Uppsala University, Uppsala, Sweden; 3 Institute for Translational Medicine and Biotechnology, Sechenov First Moscow State Medical University, Moscow, Russia

**Keywords:** Neuroblastoma, Differentiation, SV2A, SV2B, SV2C, Synaptic vesicle protein

## Abstract

**Background:**

The synaptic vesicle glycoprotein 2 (SV2) family is essential to the synaptic machinery involved in neurotransmission and vesicle recycling. The isoforms SV2A, SV2B and SV2C are implicated in neurological diseases such as epilepsy, Alzheimer’s and Parkinson’s disease. Suitable cell systems for studying regulation of these proteins are essential. Here we present gene expression data of *SV2A*, *SV2B* and *SV2C* in two human neuroblastoma cell lines after differentiation.

**Methods:**

Human neuroblastoma cell lines SiMa and IMR-32 were treated for seven days with growth supplements (B-27 and N-2), all-*trans*-retinoic acid (ATRA) or vasoactive intestinal peptide (VIP) and gene expression levels of *SV2* and neuronal targets were analyzed.

**Results:**

The two cell lines reacted differently to the treatments, and only one of the three SV2 isoforms was affected at a time. *SV2B* and choline O-acetyltransferase (*CHAT)* expression was changed in concert after growth supplement treatment, decreasing in SiMa cells while increasing in IMR-32. ATRA treatment resulted in no detected changes in *SV2* expression in either cell line while VIP increased both *SV2C* and dopamine transporter (*DAT)* in IMR-32 cells.

**Conclusion:**

The synergistic expression patterns between *SV2B* and *CHAT* as well as between *SV2C* and *DAT* mirror the connectivity between these targets found in disease models and knock-out animals, although here no genetic alteration was made. These cell lines and differentiation treatments could possibly be used to study SV2 regulation and function.

## Introduction

There has been growing interest in the three members of the synaptic vesicle glycoprotein 2 (SV2) family driven by connections to diseases. Mutation in the human *SV2A* gene is linked to epilepsy [1], and the loss of SV2A causes early postnatal lethality in mice due to severe seizures [2, 3], impaired neurotransmission [4, 5] and defects in the trafficking of the calcium-sensing vesicular protein synaptotagmin 1 [6]. The functional role of SV2B, an isoform less ubiquitously expressed than SV2A [7, 8] that likewise interacts with synaptotagmin 1 [9], is unclear. SV2B knock-out (KO) mice do not suffer from seizures [3]; however, they are protected from amyloid-β_25-35_ peptide induced toxicity typically causing memory and cholinergic deficit [10]. SV2C has the most restrictive expression pattern [11, 12] of the three isoforms. It is expressed in some GABAergic neurons, especially the Purkinje cells of the cerebellum, some dopaminergic neurons and in a fraction of cholinergic neurons [13]. *SV2C* mRNA levels are elevated in Parkinson’s disease (PD) models [13], and SV2C KO mice present up-regulation in tyrosine hydroxylase mRNA [12], the rate limiting enzyme in dopamine synthesis [14]. PD patients display more SV2C-positive labeled neurons than normal aged brains [15] while a decrease of SV2C is found in models for Huntington’s disease [16]. In addition, SV2A, SV2B and SV2C all bind botulinum neurotoxins (BoNTs) [17] and allow an entryway for the toxins into neurons via vesicles as the SV2 proteins are internalized. BoNTs, perhaps most known for the botulinum neurotoxin type A (BoNT/A) breakthrough in the aesthetics industry, also inhibit growth of human prostate cancer cells [18] and alter the expression of SV2 in breast cancer cell lines [19]. The expression patterns and regulation of SV2 proteins in different settings are becoming increasingly important to understand as they function in an essential part of neurotransmitter release, are altered in several diseases and can be utilized as drug targets.

Here we have analyzed the alterations of *SV2A*, *SV2B* and *SV2C* in two human neuroblastoma cell lines, SiMa and IMR-32, after treatment with B-27 and N-2 Supplements (B27/N2), all-*trans* retinoic acid (ATRA) and vasoactive intestinal peptide (VIP). Serum-free growing conditions supplemented with B27/N2 have previously been used to increase the effect of BoNT/A in SiMa cells [20]. While the improvement was clear, the reason was not pin-pointed and could be due to general maturation of the cell line, increase in SV2 receptor proteins [21] or an increase in the intracellular synaptosomal-associated protein 25 (SNAP25), the target of BoNT/A [22]. Retinoid, a metabolite of vitamin A, is vital for development from embryogenesis to adulthood [23, 24], and inhibits the growth of tumor cell lines, including many neuroblastoma cell lines [24, 25]. Retinoic acid (RA) isoforms exert diverse effects in different cell lines [26, 27] and both 9-*cis* and ATRA are used in vitro to induce apoptosis or differentiation in neuroblastoma cell lines. VIP, a neuropeptide with a wide distribution in the body, regulates proliferation and differentiation in both normal and tumorous cells [28, 29]. In addition, VIP inhibits inflammatory pathways [30], common in neurodegenerative disorders such as PD, possibly by reducing oxidative stress [31]. The effects of *SV2A*, *SV2B* and *SV2C* expression, previously not monitored under these conditions, were here analyzed together with a selection of neuronal markers: choline O-acetyltransferase (*CHAT*) as a marker for cholinergic neurons [32], dopamine transporter (*DAT*, *SLC6A3*) as a marker for dopaminergic neurons [33] and norepinephrine transporter (*NET*, *SLC6A2*) to monitor neuroadrenergic neurons [34]. *SNAP25* as an indication of neurite outgrowth [35] and fibroblast growth factor receptor 3 (*FGFR3*) as an additional receptor for BoNT/A [36] were used. Both cell lines reacted in different ways, indicating that the SV2 proteins are intricately and differentially regulated in different neuroblastoma cells. Intriguingly, co-regulation was seen between *SV2B* and *CHAT*, and *SV2C* and *DAT*, after some of the treatments, reflecting a possible regulatory connection between these targets as the cell lines respond to the treatments.

## Material and methods

### Fluorescent immunocytochemistry of SiMa and IMR-32 cells

Immunocytochemistry (ICC) was performed as described by Perland et al. [37] on wild-type (wt) undifferentiated SiMa and IMR-32 cells grown in the media recommended by DSMZ (see below), fixed using 4% PFA (Histolab) for 60 min and then washed in PBS 3 × 10 min. Cells were again washed in PBS 3 × 10 min, blocked in Supermix blocking solution (0.25% gelatin, 0.5% Triton X-100 in TBS) for 1 h, and stained with primary antibodies overnight, all diluted in Supermix blocking solution; SV2A (HPA007863, Sigma-Aldrich or AV47093) diluted 1:100, SV2B (SAB1406892 for SiMa cells, HPA046247 for IMR-32 cells, Sigma-Aldrich) diluted 1:100, SV2C (HPA040722 Sigma Aldrich), SNAP25 (S9684, Sigma-Aldrich) diluted 1:200, Beta-tubulin (Abcam, RRID: SCR_012931) diluted 1:200, Syntaxin 1a (Ab13262, Abcam) diluted 1:200, and Pan neuronal cocktail (MAB2300, Millipore) diluted 1:200. Several antibodies for SV2 targets were tested during ICC optimization. Secondary antibodies, Alexa Fluor, were all purchased from Invitrogen and diluted 1:800. DAPI (Sigma-Aldrich) was diluted 1:10,000 in PBS. Images were acquired at the SciLifeLab BioVis Facility (Uppsala University) with a Zeiss LSM710 Confocal microscopy and the Zen software (Zeiss). Stacks were merged and handled using ImageJ (Fiji edition, RRID: SCR_002285) [38].

### Identification of predicted transcription factor recognition motifs

Using the Eukaryotic Promoter Database (http://epd.vital-it.ch/) [39, 40], we predicted the presence of transcription factor motifs 1000 base pairs (bp) up-stream of the transcription start site (TSS) for the human genes *SV2A* (Ensemble ID: ENSG00000159164), *SV2B* (Ensemble ID: ENSG00000185518) and *SV2C* (Ensemble ID. ENSG00000122012). Motifs were chosen from the Transcription Factor Motifs (JASPAR CORE 2016 vertebrates) library and possible hits with a cut-off p-value of at least 0.001 or lower were used.

### Cell culture conditions

Reagents and material are from Thermo Fisher Scientific if not stated otherwise. SiMa cells (ACC 164, German Collection of Microorganism and Cell Cultures, DSMZ, RRID: SCR_001711) were cultured in RPMI 1640 medium 2 mM GlutaMAX, 1× NEAA, 1× Pen-Strep and 10% fetal bovine serum. IMR-32 cells (ACC 165, German Collection of Microorganism and Cell Cultures, DSMZ, RRID: SCR_001711) were cultured in RPMI 1640 medium, 2 mM GlutaMAX, 1× NEAA, 1× Pen-Strep and 20% fetal bovine serum, according to the recommendations of the cell provider. Cells were grown on Nunclon Delta treated petri dishes and incubated at 37 °C with 5% CO_2_. For the differentiation experiments the cells were grown to 60% confluence in 24-well Nunclon Delta plates. All three treatments were performed for a total of seven days with a 50% change of media every second day and each treatment was performed with its own control. Growth supplements using B-27 and N-2 serum-free Supplements (referred to as B27/N2 treatment) were added to complete media (as described above) without FBS. Controls were cultured in complete media without FBS and supplement. ATRA (R2625, Sigma-Aldrich) was diluted in DMSO (Sigma-Aldrich) to a stock solution of 30 mg/mL and kept at -80 °C. For each change of media, the stock solution was further diluted in 70% ethanol to 0.3 mg/mL and added to complete media with 50% reduced FBS concentration, to a concentration of 1 µM. Control media were prepared using DMSO diluted in 70% ethanol. VIP (V6130, Sigma-Aldrich) was diluted in 1% acetic acid (Sigma-Aldrich) to 250 µg/mL and then diluted to 1 µM with complete media with 50% reduced FBS concentration. Controls were treated with 1% acetic acid added to the media in the same volumes as in the treatment group. The B27/N2 treatment was hence performed in serum-free conditions, while the ATRA and VIP were performed under serum-reduced conditions to be easier to compare with other differentiation procedures previously performed.

### RNA preparation and cDNA synthesis

Five wells per experimental group (n = 5 treated and n = 5 control) were used for RNA extraction. The RNA was retrieved using Allprep DNA/RNA micro kit (Qiagen), according to the manufacturer’s instructions. Concentrations were measured using a NanoDrop 1000 spectrophotometer (NanoDrop Technologies). cDNA synthesis, with an Applied Biosystems High Capacity RNA-to-cDNA kit (Invitrogen), was performed using 2 µg of RNA template according to the manufacturer’s recommendations. cDNA concentration was measured using an ND-1000 spectrophotometer and diluted to 30 ng/µL with sterile nuclease-free water.

### Primer design and quantitative real-time PCR (qRT-PCR)

Gene expression changes were determined using qRT-PCR. All primers were designed using Beacon Design 8 (Premier Biosoft), Table 1. The final volume for each qRT-PCR reaction was 20 µL, consisting of: 3 µL cDNA, 0.05 µL of each primer (100 pmol/µL), 3.6 µL of 10× DreamTaq buffer (Thermo Fisher Scientific), 0.2 µL of 25 mM dNTP mix (Thermo Fisher Scientific), 1 µL of DMSO, 0.5 µL of SYBR Green diluted 1:10,000 (Invitrogen) and 0.08 µl of Dream Taq (5 U/µl, Thermo Fisher Scientific). Volumes were adjusted to 20 µL with sterile water. CFX96 connect (Bio-Rad Laboratories, RRID:SCR_008426) was used with the following settings: initial denaturation for 30 s at 95 ºC, 55 cycles of 10 s at 95 ºC, 30 s at 55–61 ºC (optimal temperature depending on primer) and 30 s at 72ºC. The melting curve was performed starting at 55 ºC for 81 cycles at 10 s intervals and a temperature increase of 0.5 ºC per cycle. All qRT-PCR were run in triplicate and a negative control was included on each plate. All data were collected using the CFX maestro (Bio-Rad Laboratories) software.

**Table 1:** List of primers used for qRT-PCR reactions

Target	Forward primer	Reverse primer	Annealing temp (°C)
RPL13A*	5ʹ-cctggaggagaagaggaaagaga-3ʹ	5ʹ-ttgaggacctctgtgtatttgtcaa-3ʹ	57.1
RPL19*	5ʹ-tgaggagaatgaggattt-3ʹ	5ʹ-gtacaggctgtgatacat-3ʹ	54.1
H3F3B*	5ʹ-cattatctaggtccttgta-3ʹ	5ʹ-aatacagcactattatgg-3ʹ	54.1
B2M*	5ʹ-gactggtctttctatctct-3ʹ	5ʹ-cttcaaacctccatgatg-3ʹ	55
HPRT1*	5ʹ-aagcctaagatgagagtt-3ʹ	5ʹ-ccacagaactagaacattg-3ʹ	55.8
GAPDH*	5ʹ-cctcaagatcatcagcaat-3ʹ	5ʹ-ttccacgataccaaagtt-3ʹ	55
TUBB3*	5ʹ-ggcatctcttgagaacaa-3ʹ	5ʹ-gacctgtacctgtctctc-3ʹ	55
*SV2A*	5ʹ-agcatgacgaaggtgaat-3ʹ	5ʹ-gtagctgtgacgtgttgta-3ʹ	58.9
*SV2B*	5ʹ-ttcaggcactaaggtctct-3ʹ	5ʹ-aaggaagcgattctcaatgtt-3ʹ	57.1
*SV2C*	5ʹ-ctggagcgttctgttttg-3ʹ	5ʹ-aattttcacagcctcctttc-3ʹ	57.1
*DAT*	5ʹ-gccgtggtcttcttcatcat-3ʹ	5ʹ-aacagggacaggaggaaggt-3ʹ	58.1
*CHAT*	5ʹ-agatgttctgctgctatg-3ʹ	5ʹ-gaaaaggatggtctctgg-3ʹ	55.8
*NET*	5ʹ-agcttttggtggtcagatgg-3ʹ	5ʹ-cagagcaagagcagcatgag-3ʹ	61.4
*SNAP25*	5ʹ-taacacttcttacgcaatg-3ʹ	5ʹ-gaacgaaccaactgatt-3ʹ	57.1
*FGFR3*	5ʹ-agactgaaattacgggta-3ʹ	5ʹ-ccatatacacagcatctatt-3ʹ	55.8

Primers used for housekeeping genes are marked with an asterisk (*)

### Analysis of qRT-PCR data

Primer efficiency for each primer was calculated using LinRegPCR software, followed by Grubbs’ test (GraphPad software) to remove outliers before Ct mean values were corrected. Due to the different characteristics of the two cell lines, and the different treatments used on them, several different housekeeping genes were screened to find several markers that were stable during the differentiation. The GeNorm protocol [41] was used to find stable housekeeping genes, and the expression was normalized using the Geomean from the stable housekeeping genes. In total, five stable housekeeping genes were found for SiMa: *GAPDH*, *TUBB3*, *B2M*, *RPL13A*, and *RPL19*, while four genes were found for IMR-32 cells: *GAPDH*, *HPRT*, *H3F3B*, and *RPL19*. Relative mRNA expression was plotted for each gene using bar plots of average mRNA expression and error bars indicating standard error of the mean (SEM). Statistical analysis was performed in GraphPad Prism version 5 (RRID: SCR_002798). T-tests were performed for gene expression changes compared to controls and significant limits set to *p < 0.05, **p < 0.01, ***p < 0.001. For visualization of gene regulation the controls for each target in each cell line were set to 100%.

## Results

**Fig. 1:**
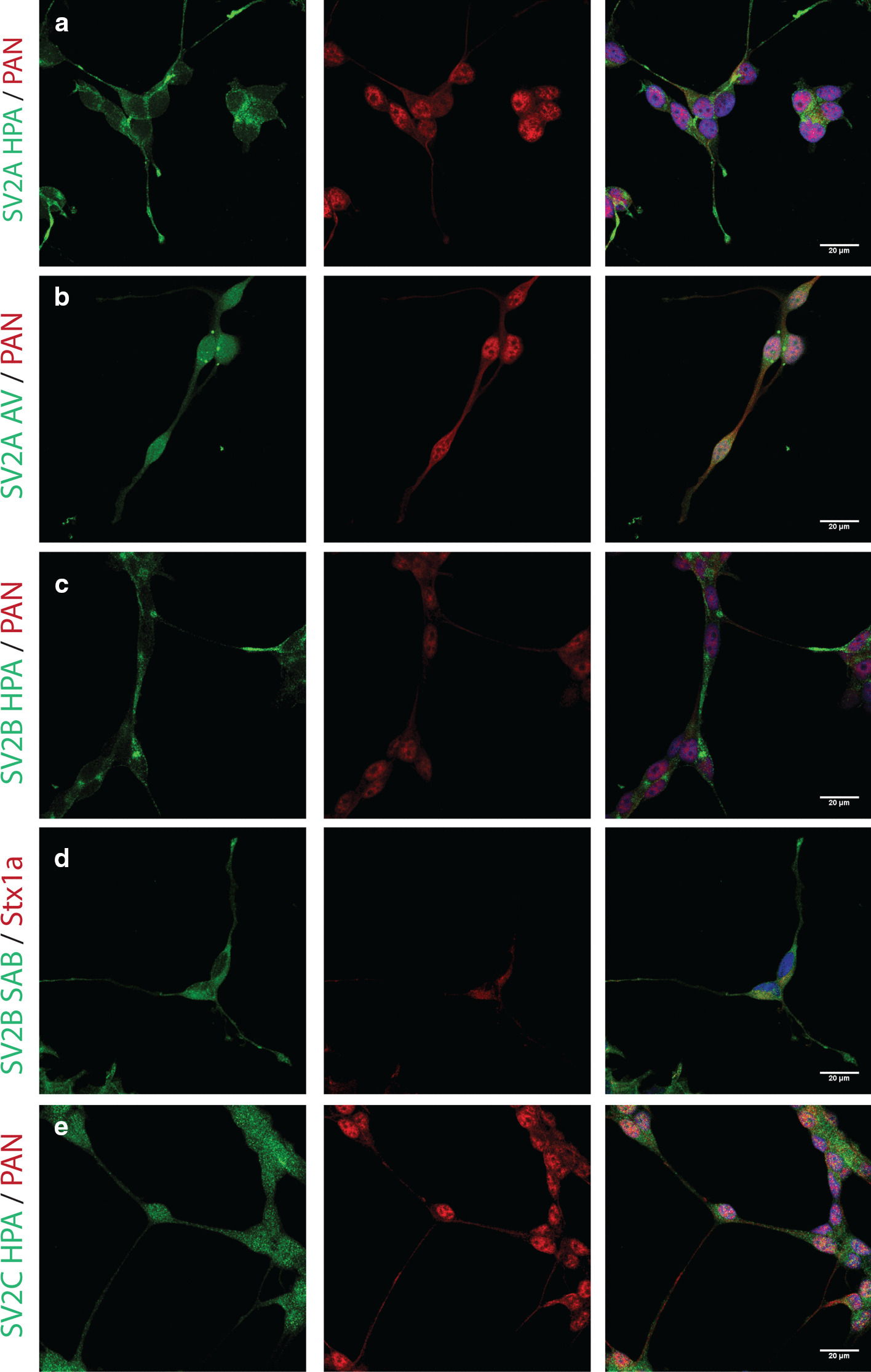
Immunocytochemistry of SiMa SV2 proteins. SV2 isoforms were stained in green, Pan neuronal cocktail in red, nuclear staining using DAPI depicted in blue. Images acquired using confocal microscopy, merged images of three stacks. Scale bar 20 µm. Several antibodies were used for the SiMA cell lines. **a**, **b** SiMa cells stained for SV2A (green) using two different antibodies and neuronal marked (red) reveal punctuate SV2A staining in processes and around soma. **c**, **d** SiMa cells stained for SV2B (green) using two different antibodies and neuronal marker (red) or Stx1a (red) reveal moderate staining in projections. **e** SiMa cells stained for SV2C (green) and neuronal marked (red) reveal staining in the cell soma

**Fig. 2:**
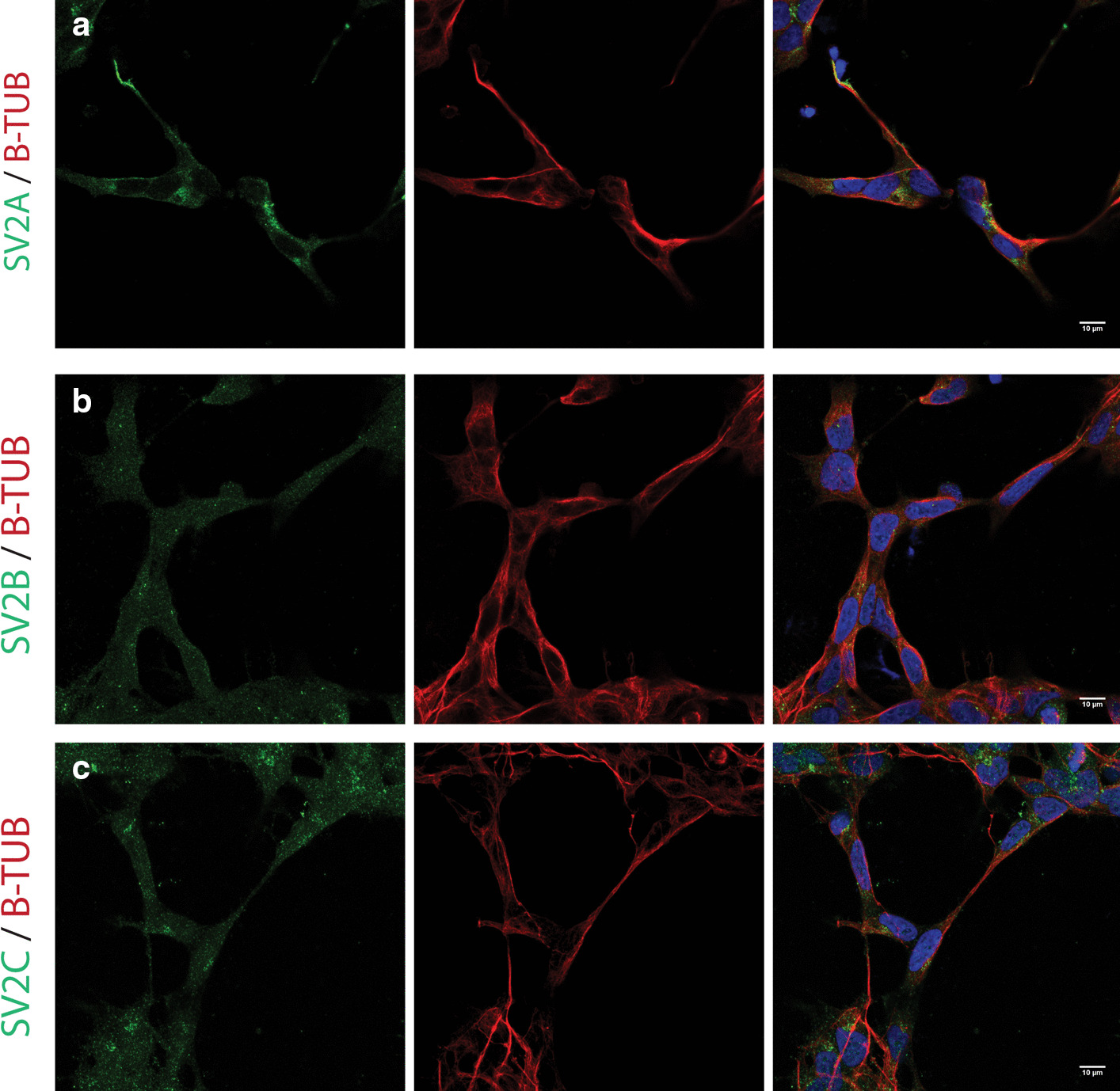
Immunocytochemistry of IMR-21 cells for SV2 proteins. SV2 isoforms were stained in green, Beta-tubulin in red, nuclear staining using DAPI depicted in blue. Images acquired using confocal microscopy, merged images of six stacks for IMR-32 cells. Scale bar 10 µm. **a** IMR-32 cells stained for SV2A (green) and B-Tub (red) reveal SV2A staining in projections. **b** IMR-32 cells stained for SV2B (green) and B-Tub (red) reveal low levels of SV2B staining. **c** IMR-32 cells stained for SV2C (green) and B-Tub (red) reveal punctuate SV2C staining in some parts of the cells

The usefulness of the two picked human neuroblastoma cell lines for studying SV2 expression patterns was initially probed using immunocytochemistry. Both neuroblastoma cell lines stained positive for SV2A, while faint to moderate staining was seen for SV2B and SV2C in their un-differentiated state. Several antibodies were tested for ICC producing staining correlating with previously published data and without background staining. The staining appeared in a punctuated pattern, as expected from vesicular proteins and in line with previously published results [42, 43], Figs. 1 and 2.

### Promoter regions upstream of SV2A, SV2B and SV2C reveal possible binding sites for transcription factors important in ATRA and VIP regulation

Three different protocols were used for differentiation of the two cell lines: B-27 and N-2 supplements (B27/N2), all-*trans* retinoic acid (ATRA) and vasoactive intestinal peptide (VIP). The effect of additives on differentiation or apoptosis is dependent on both interaction with receptors and the presence of transcription factor motifs capable of activating or inhibiting gene expression. Using the Eukaryotic Promoter Database (http://epd.vital-it.ch/) [39, 40] we searched for predicted transcription factor binding motifs present in the region 1000 bp upstream of the transcription start site (TSS) of *SV2A*, *SV2B* and *SV2C*. Both B27 and N2 Supplement consist of a cocktail of substances and the concentration of additives in the B27 Supplement is proprietary, making the relevant transcription factor motifs harder to predict than for ATRA and VIP. Both B27 Supplement and ATRA contain vitamin A (retinoic acid, RA) and gene activation is dependent on presence of retinoic acid response elements (RARE) in the promoter region where retinoic acid receptors (RAR) or retinoid X receptor (RXR) can bind [44, 45]. VIP exerts its effect through three different receptors, which results in diverse signaling cascades, many converging in cAMP production [46–48]. Based on the prediction of the Eukaryotic Promoter Database, all SV2 isoforms contain motifs for transcription activation by RA and VIP. *SV2A* holds four possible sites for activation by ATRA, and four possible sites for VIP via the cAMP response element binding protein (CREB), Table 2, while *SV2B* contains nine for ATRA and three for CREB. In the upstream region of *SV2C*, two possible ATRA binding sites were found and one for CREB, with an additional one at 92 pb upstream of the TSS.

**Table 2:** Localization of gene activation sites upstream of transcription start sites of human genes *SV2A*, *SV2B* and *SV2C*

Transcription factor motif	Location upstream of *SV2A*	Location upstream of *SV2B*	Location upstream of *SV2C*
RARα	363 bp	242 bp 284 bp 645 bp	No predicted sites
RARα v.2	194 bp 330 bp 991 bp	54 bp 427 bp 486 bp 569 bp 797 bp 890 bp	255 bp 332 bp
CREB1	No predicted sites	459 bp 564 bp 952 bp	92 bp
CREB3	878 bp	No predicted sites	128 bp
CREB3L1	282 bp 576 bp 956 bp	No predicted sites	No predicted sites

Predictions were made using the Eukaryotic Promotor Database using motifs from the Transcription Factor Motifs library and possible hits with a cut-off p-value of at least 0.001 were used. Predictions were made of an area of 1000 base pairs (bp) upstream from the transcription start site

### SiMa cell line alters SV2 expression after seven days of B27/N2, but not after ATRA and VIP treatment

To be able to keep the cells healthy for seven days without sub-culturing them, all three differentiation protocols were performed in combination with serum deprivation. The control cells, not receiving the additives tested, were hence also serum deprived to allow a just comparison. Serum deprivation in and of itself can be used as a differentiation protocol [49]. However, our goal was to look at the effect of the different additives: B27/N2 supplement, ATRA and VIP. The differentiation with B27/N2 was done together with complete serum starvation, while that of ATRA and VIP was performed using a 50% reduction of serum compared to normal culture conditions for each cell line. Due to biological differences between the cell lines used and the differences in supplements tested here, a larger panel of housekeeping genes was tested to make certain that stable markers were used to perform the gene expression normalization against. Five stable genes were found for the SiMa cell line that could be used for normalization for all of the treatments, while four were found for the IMR-32 cell line; see “Materials and methods” section.

**Fig. 3:**
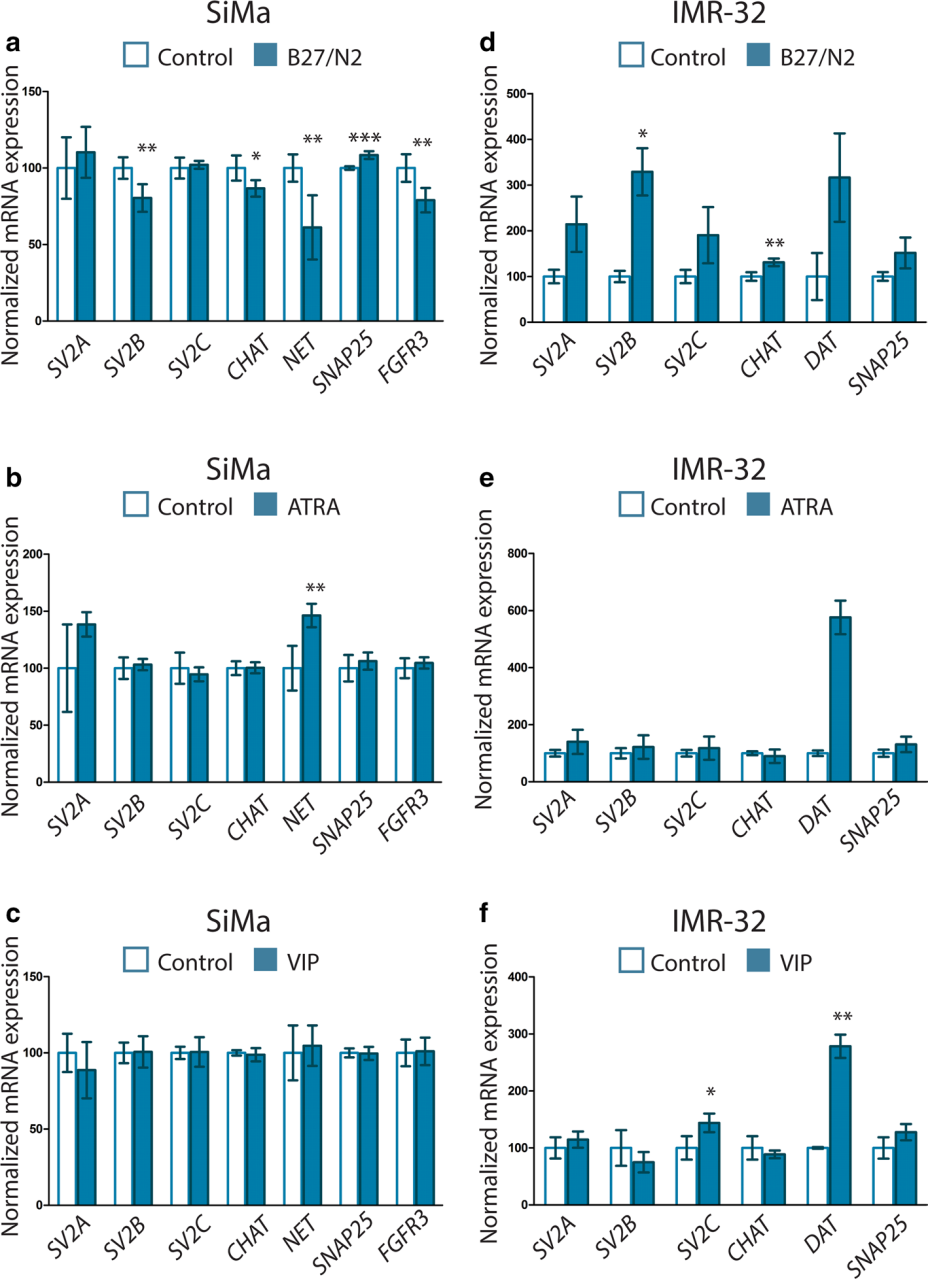
Gene expression changes in SiMa and IMR-32 cell line differentiated for seven days. The mRNA expression was first normalized using *GAPDH*, *TUBB3*, *B2M*, *RPL13A* and *RPL19* for the SiMa cell line and *GAPDH*, *HPRT*, *H3F3B* and *RPL19* for IMR-32 cells. Bar diagram over average relative mRNA expression level with error bars indicating standard error of the mean (SEM). Controls (open barn) set to 100% for each target, and expression in treated samples (colored bars) compared to each control. T-tests were performed for gene expression changes between five biological replicates for control (n = 5) and treated cells (n = 5), where *p < 0.05, **p < 0.01, ***p < 0.001. **a** SiMa cells cultured in serum-free media with B27/N2. Altered gene expression was seen for *SV2B* (p = 0.0047), *CHAT* (p = 0.0230), *NET* (p = 0.0011), *SNAP25* (p = 0.0004) and *FGFR3* (p = 0.0063) compared with cells cultured in serum-free media. **b** SiMa cells cultured in serum-reduced media supplemented with 1 µM all-*trans*-retinoic acid (ATRA). Altered gene expression could be found for *NET* (p = 0.0058). **c** SiMa cells cultured in serum-reduced media with 1 µM vasoactive intestinal peptide (VIP). No alterations in gene expression were found compared with vehicle controls. **d** IMR-32 cells cultured in serum-free media with B27/N2. Altered gene expression was seen for *SV2B* (p = 0.0332), and *CHAT* (p = 0.0050) compared to cells cultured in serum-free media. **e** IMR-32 cells cultured in serum-reduced media supplemented with 1 µM all-*trans*-retinoic acid (ATRA). Here, n = 4 for each group was used. No gene alterations were seen for this treatment compared with vehicle controls. **f** IMR-32 cells cultured in serum-reduced media with 1 µM vasoactive intestinal peptide (VIP). Altered gene expression was seen for *SV2C* (p = 0.0227), and *DAT* (p = 0.0019) compared with cells cultured in serum-free media

For the SiMa cell line, the addition of B27/N2 Supplement produced the most alterations after the seven-day treatment and VIP treatment the least. Seven days of serum starvation together with B27/N2 Supplement treatment decreased *SV2B* mRNA levels (p = 0.0047), Fig. 3a. In addition, *CHAT* (p = 0.0230), *NET* (p = 0.0011) and *FGFR3* (p = 0.0063) gene expression levels were all decreased compared with controls while *SNAP25* gene expression was slightly increased (p = 0.0004). Seven days of serum reduction and 1 µM ATRA treatment resulted in an increase of only *NET* (p = 0.0058), Fig. 3b. Treatment using 1 µM VIP produced no change in expression, Fig. 3c, in the SiMa cell line.

### IMR-32 cell line increased SV2 expression after both B27/N2 and VIP treatment, while ATRA produced no alterations.

Overall, the gene expression of the treated group for all of the three different protocols was more variable than the control cells, indicating a more biologically heterogeneous population of cells where some respond to the treatment and some do not. The gene expression was also more variable than that of the SiMa cells. IMR-32 subjected to serum starvation and B27/N2 for seven days increased both *SV2B* mRNA expression (p = 0.0332) and that of *CHAT* (p = 0.0050), Fig. 3d. No mRNA alterations for the targets tested could be seen to occur after seven days of ATRA treatment, Fig. 3e. Seven days of serum reduction and 1 µM VIP treatment resulted in up-regulation of *SV2C* mRNA expression (p = 0.0227) accompanied by an increase in *DAT* mRNA expression (p = 0.0019), Fig. 3f.

## Discussion

Understanding the regulation of SV2 proteins in different biological settings is becoming essential due to their involvement in vesicular trafficking, diseases and drug delivery [4, 22, 50, 51]. Their gene expression in two human neuroblastoma cell lines, SiMa and IMR-32, was here monitored after three different treatments—serum starvation with B27/N2, serum deprivation with ATRA, and serum deprivation with VIP—to study changes in *SV2* expression and to gain insight into whether these cell lines could be valuable in the study of *SV2* alterations. These targets have not been studied before under these conditions, even though these treatments have previously been used on neuroblastoma cell lines where previous studies indicate that differentiation of neuroblastoma cell lines is highly variable and cell line specific [27, 52]. A seven-day treatment time was chosen based on previous studies but also with the goal of capturing more long lasting, and possibly more stable, alterations. Neuroblastoma cells subjected to RA and VIP for a short time (0.5 h–96 h) reveal fluctuating levels of proto-oncogene MYCN over the time-course of the experiment [53], indicating a too dynamic time period. A screen of several RA differentiated neuroblastoma cell lines revealed seven days to be an optimal time for the proto-oncogene *RET* response in IMR-32 cells [27] and long differentiation times steadily increase neurite growth for some neuroblastoma cell lines treated with ATRA [54]. Both RA and VIP differentiation has commonly been performed under reduced serum conditions, and as the recommended serum concentrations were different for the two cell lines under normal culture conditions, the RA and VIP treatment was done using a 50% serum reduction, providing a more similar alteration in serum levels as opposed to a numerically equal one. The B27/N2 treatment was however performed with no serum for both cell lines, and both cell lines were found to handle this serum starvation well. It is possible that the differentiation protocols for RA and VIP could have been enhanced using no serum during treatment. However, the gene alterations seen after the treatments are compared to controls cultured with the same serum levels and additives as the treatment group but without the addition of B27/N2, RA or VIP, and hence represent the alterations due to the differentiation agents used.

The seven-day treatments used here elicited different responses in both cell lines. The expression levels observed in SiMa cells were generally higher even than those found in the IMR-32 cells. This could be attributed to the heterogeneous characteristics of IMR-32 cells. According to records available at the European Collection of Authenticated Cell Cultures (ECACC) as well as the American Type Culture Collection (ATCC), the IMR-32 cell culture is a mixture of two distinct cell types, one more predominant, smaller and neuroblast-like cell type and the other hyaline fibroblast like. In addition, the genes that were affected by treatment in the IMR-32 cells were found to undergo larger changes than in the SiMa cells. After B27/N2 treatment, for example, both cell lines had altered *SV2B* expression, with a change of 20% compared to controls in the SiMa cell line while IMR-32 cells had an increase in expression of 228% as compared to controls. Likewise, the expression of *DAT* after VIP treatment of IMR-32 cells rose by almost 180% compared to controls. Perhaps the heterogeneity of IMR-32 cells does add more variability to the gene expression data, but the responsive cells in the cultures were more responsive than the SiMa cells. In our setting, none of the treatments led to simultaneous up-regulation or down-regulation of two or more SV2 isoforms. This has however been observed by others. An effect on all three SV2 isoforms in non-human cell lines Neuro2-a and PC12, after two to three days of B27/N2 treatment, has been reported [36] in a set up used to increase the sensitivity to and potency of BoNT/A [20]. In our setting, B27/N2 differentiation of SiMa cells resulted in an increase of *SNAP25*, while no effect was seen on *SV2C*. SV2C is the main receptor of BoNT/A and SNAP25 is the SNARE protein target for its enzymatic activity [55]. Furthermore, *FGFR3*, with the protein product FGFR3 found to be an additional receptor of BoNT/A [36], was decreased. The SiMa cell line has been used in BoNT/A research and proven sensitive to the neurotoxin [20]; however, an improved uptake or easier readout of the proteolytic activity of BoNT/A would still be beneficial. The longer treatment time used here might not be optimal for increased sensitivity due to the decrease of important BoNT/A receptors. The B27/N2 treatment produced interesting synergistic effects for the expression of *SV2B* and *CHAT*, Fig. 3a, d; both decreased in the SiMa cell line while both increased in the IMR-32 cells. SV2B KO mice subjected to toxic species of amyloid-β protein are protected from both memory decline and decrease in *CHAT* activity in the hippocampus [10], opening up the possibility that *SV2B* is needed to regulate *CHAT.* Furthermore, reduced SV2B expression has also been found in other neurodegenerative diseases connected to acetylcholine [56–58]. The co-regulation between *SV2B* and *CHAT* in the neuroblastoma cell lines used here indicates that interconnectivity exists between SV2B and CHAT even outside of disease models where SV2B is missing, or due to failure in cholinergic neurons. Evidence of a physical interaction between SV2B and CHAT is not established according to the BioGrid database of protein, genetic and chemical interactions [59] and the co-regulations seen in disease models and here are likely due to pathway alterations modifying a response upstream of both *SV2B* and *CHAT.*

Different isoforms of RA, 9-*cis* RA or ATRA, cause different modifications in RA-sensitive cell lines, ranging from apoptotic for the SK-N-FI cell line to differentiation for the SH-SY5Y cell line [27]. After the ATRA treatment here, no change in *SV2* expression was found for either cell line. The effects of ATRA rely on activation of retinoic responsive elements (RAREs), and based on our predictions, all three SV2 genes could possess transcription activation binding sites. The IMR-32 cell line has previously been treated with both 9-*cis* RA and ATRA, and gene expression profiling of apoptotic markers revealed an increase of oncogene *RET* level after seven days of treatment by both forms of RA, indicating the capability of RA to induce differentiation [27], but the effect on SV2 proteins was not studied. ATRA produced a large gene expression spread in the treated IMR-32 cells compared to controls. This again indicates that the IMR-32 cell culture is heterogeneous, containing some cells that respond to the treatment while some do not, which has been seen in IMR-21 cells previously [60].

Based on the targets monitored in this study, VIP had no effect on the SiMa cells, possibly due to the lack of VIP receptors, VPAC1 and VPAC2 [61], or on the low copy number of MYCN, which is proposed to be the reason why a similar neuroblastoma cell line, SH-SY5Y, does not react to VIP [53]. In contrast, an increase of both *SV2C* and *DAT* was seen in the IMR-32 cells. SV2C is the isoform with the most restrictive expression pattern in the mouse brain [12, 13, 62], with particularly high expression in several basal ganglia nuclei [12]. In the mouse brain, SV2C can be found in dopaminergic neurons and to a lesser extent in cholinergic neurons [13]. Variations in the *SV2C* gene are linked to predictions in PD patients’ sensitivity to L-DOPA [63] and were found to regulate both dopamine release and content. The co-regulation of *SV2C* and *DAT* seen here after VIP treatment fits the already established connection in disease models; however, here no alterations were made directly towards *SV2C* or genes in the dopamine pathway. Similarly as in the case of *SV2B* and *CHAT*, no physical interaction can be found for *SV2C* and *DAT* in the BioGrid database [59].

## Conclusion

*SV2* gene expression has not previously been monitored in these two human neuroblastoma cell lines and the alterations due to the treatments indicate that the *SV2* expression is affected by factors in the B27/N2 growth supplements and VIP, but not by ATRA. A synergistic effect was found for *SV2B* and *CHAT* in both cell lines, a connection previously shown in disease models for AD. In addition, *SV2C* and *DAT* were up-regulated after VIP treatment in IMR-32 cells, corroborating a connection between SV2C and dopamine seen in PD models and patients. The co-regulations seen here, in non-disease models, verify the importance and co-dependence of these targets and could indicate that these treatments and cell lines could be suitable for the study of SV2 regulation and function.

## Data Availability

The datasets used and analyzed during the current study are available from the corresponding author on reasonable request.
